# Supplementary material for: Deep learning for detecting retinal detachment and discerning macular status using ultra-widefield fundus images
Source: Commun Biol. 2020 Jan 8;3:15. doi: 10.1038/s42003-019-0730-x (PMC6949241; doi:10.1038/s42003-019-0730-x)
Supplement: Supplementary file 2 — Reporting Summary [file 42003_2019_730_MOESM2_ESM.pdf]

nature research

Corresponding author(s): Haotian Lin

Last updated by author(s): 2019-11-25

Reporting Summary

Nature Research wishes to improve the reproducibility of the work that we publish. This form provides structure for consistency and transparency in reporting. For further information on Nature Research policies, see Authors & Referees and the Editorial Policy Checklist.

Please do not complete any field with "not applicable" or n/a. Refer to the help text for what text to use if an item is not relevant to your study. For final submission: please carefully check your responses for accuracy; you will not be able to make changes later.

Statistics

For all statistical analyses, confirm that the following items are present in the figure legend, table legend, main text, or Methods section.

n/a	Confirmed	
☐	☒	The exact sample size (*n*) for each experimental group/condition, given as a discrete number and unit of measurement
☐	☒	A statement on whether measurements were taken from distinct samples or whether the same sample was measured repeatedly
☐	☒	The statistical test(s) used AND whether they are one- or two-sided<br>*Only common tests should be described solely by name; describe more complex techniques in the Methods section.*
☒	☐	A description of all covariates tested
☒	☐	A description of any assumptions or corrections, such as tests of normality and adjustment for multiple comparisons
☐	☒	A full description of the statistical parameters including central tendency (e.g. means) or other basic estimates (e.g. regression coefficient) AND variation (e.g. standard deviation) or associated estimates of uncertainty (e.g. confidence intervals)
☐	☒	For null hypothesis testing, the test statistic (e.g. *F*, *t*, *r*) with confidence intervals, effect sizes, degrees of freedom and *P* value noted<br>*Give P values as exact values whenever suitable.*
☒	☐	For Bayesian analysis, information on the choice of priors and Markov chain Monte Carlo settings
☒	☐	For hierarchical and complex designs, identification of the appropriate level for tests and full reporting of outcomes
☒	☐	Estimates of effect sizes (e.g. Cohen's *d*, Pearson's *r*), indicating how they were calculated

Our web collection on statistics for biologists contains articles on many of the points above.

Software and code

Policy information about availability of computer code

Data collection	No software was used.
Data analysis	We used a receiver operating characteristic (ROC) curve and the area under the curve (AUC) with 95% confidence intervals (CI) to evaluate the performance of the DL models. The accuracy, sensitivity, and specificity of the DL models and general ophthalmologists for detecting RD and discerning the macular status were computed according to the reference standard. In the test sets, unweighted Cohen's kappa coefficients were employed to compare the results of the DL models to the reference standard as determined by the aforementioned retinal specialists. All statistical analyses were performed using Python 3.7.3.

For manuscripts utilizing custom algorithms or software that are central to the research but not yet described in published literature, software must be made available to editors/reviewers. We strongly encourage code deposition in a community repository (e.g. GitHub). See the Nature Research guidelines for submitting code & software for further information.

Data

Policy information about availability of data

All manuscripts must include a data availability statement. This statement should provide the following information, where applicable:

- Accession codes, unique identifiers, or web links for publicly available datasets
- A list of figures that have associated raw data
- A description of any restrictions on data availability

The data in this study include the training dataset and the test datasets. Correspondence and requests for data materials should be addressed to HTL (haot.lin@hotmail.com).

Field-specific reporting

Please select the one below that is the best fit for your research. If you are not sure, read the appropriate sections before making your selection.

☒ Life sciences ☐ Behavioural & social sciences ☐ Ecological, evolutionary & environmental science

Life sciences study design

All studies must disclose on these points even when the disclosure is negative.

Sample size	To develop the cascaded deep learning system, a total of 11087 UWF images were retrospectively obtained from patients presenting for retinopathy examinations or undergoing a routine ophthalmic health evaluation between November 2016 and January 2019 at Shenzhen Ophthalmic Centre using an OPTOS nonmydriatic camera (OPTOS Daytona, Dunefermline, United Kingdom) and 200° fields of view. Patients underwent this examination without mydriasis.
Data exclusions	Poor-quality images were excluded from the study, and fair-quality images were excluded from the training of the DL model designed to discern macula-on RD from macula-off RD.
Replication	In the present study, the cascaded DL system included two models, each of which was trained using a state-of-the-art convolutional neural network (CNN) architecture, InceptionResNetV2. InceptionResNetV2 mimics the architectural characteristics of two previous state of the art CNNs, the Residual Network and the Inception Network. Weights pretrained for ImageNet classification were used to initialize the CNN architectures. The first model identified RD in input images. The second model further discerned macula-on RD from RD images detected by the first model. Figure 2 shows the framework of this cascaded DL system. Each DL model was trained up to 180 epochs. During the training process, the validation loss was evaluated using the validation set after each epoch and used as a reference for model selection. Early stopping was applied, and if the validation loss did not improve over 60 consecutive epochs, the training process was stopped. The model state where the validation loss was the lowest was saved as the final state of the model.
Randomization	To train the DL model, the whole dataset was randomly divided into 3 independent sets: 70% in a training set, 15% in a validation set and the remaining 15% in a test set, with no patients overlapping among these sets.
Blinding	Before the computer scientist declared the results predicted by DL models in test sets, retina specialists were masked to those DL model results.

Reporting for specific materials, systems and methods

We require information from authors about some types of materials, experimental systems and methods used in many studies. Here, indicate whether each material, system or method listed is relevant to your study. If you are not sure if a list item applies to your research, read the appropriate section before selecting a response.

Materials & experimental systems

n/a	Involved in the study
☒	☐ Antibodies
☒	☐ Eukaryotic cell lines
☒	☐ Palaeontology
☒	☐ Animals and other organisms
☐	☒ Human research participants
☐	☒ Clinical data

Methods

n/a	Involved in the study
☒	☐ ChIP-seq
☒	☐ Flow cytometry
☒	☐ MRI-based neuroimaging

Human research participants

Policy information about studies involving human research participants

Population characteristics	A total of 11087 UWF images from 7966 patients aged 10-86 years (mean age 47.5 years, 43.6% female) were labeled for retinal detachment
Recruitment	UWF images were retrospectively obtained from patients presenting for retinopathy examinations or undergoing a routine ophthalmic health evaluation between November 2016 and January 2019 at Shenzhen Ophthalmic Centre.
Ethics oversight	This study was approved by the Institutional Review Board of Zhongshan Ophthalmic Centre (IRB: 2019KYPJ107) and adhered to the tenets of the Declaration of Helsinki.

Note that full information on the approval of the study protocol must also be provided in the manuscript.

This checklist template is licensed under a Creative Commons Attribution 4.0 International License, which permits use, sharing, adaptation, distribution and reproduction in any medium or format, as long as you give appropriate credit to the original author(s) and the source, provide a link to the Creative Commons license, and indicate if changes were made. The images or other third party material in this article are included in the article's Creative Commons license, unless indicated otherwise in a credit line to the material. If material is not included in the article's Creative Commons license and your intended use is not permitted by statutory regulation or exceeds the permitted use, you will need to obtain permission directly from the copyright holder. To view a copy of this license, visit http://creativecommons.org/licenses/by/4.0/
